# Case of Retention of Placenta

**Published:** 1874-09

**Authors:** C. E. Ristine

**Affiliations:** Edgefield, Tenn.


ARTICLE III. —*Remarkable Case of Retention of Placenta.* By C. E. RISTINE, M.D., Edgefield, Tenn.

Was called at 5 A. M., 15th of July, 1870, by Mr. B., to visit his wife, whom he said had given birth to an apparently mature child on the day previous (14th), at 4 o'clock P. M., and that the afterbirth had not been delivered. Arriving at Mr. B.'s residence, found the patient lying on her back, with head and shoulders elevated. She was a blonde, 20 years old; primapara; anemic, pale, nervous; alarmed and uneasy; suffering no pain. She was attended in her confinement by a midwife. Patient has had no pain since birth of child. External abdominal examination revealed a semi-contracted condition of uterus; soreness, but no pain, on pressure. I then proceeded to make an examination per vaginam. My hand (well oiled) was introduced into vagina, and just as the fingers had reached or touched the mouth of womb she was seized with a convulsion, which lasted about two minutes after the withdrawal of my hand. Patient said she would not submit to another examination. However, I prevailed on her to permit one more, which should be conducted with great pains and care; but nevertheless the convulsion returned, more violent than the first. I then sent for chloroform, and, under its influence, I made an examination (patient had another but milder convulsion, the force of it being only partially controlled) which revealed attachment of cord. (The cord was severed about two inches from its attachment to the placenta, by the midwife, in her endeavor to deliver it.) On the right side, placenta was tightly and completely adherent. The effort to force my finger beneath its margin caused the patient to scream aloud. Her husband, mother and sister then interfered, and begged me to desist—to withdraw my hand and let her die, if it, remaining there, would produce death.

I used no more manual effort to extract it, and, being at a loss to know what else to do, I prescribed: ergot and quinine; rest, in horizontal position; stimulating embrocations to abdomen, over region of womb; and, when admissible, the injection of warm water per vaginam.

Things continued in the same condition; no alarming symptoms for twenty-eight days, or until the 11th of August. At 12 o'clock M. on that day the patient had several severe pains, the last one expelling the placenta, to the great satisfaction and extreme joy of the patient and relatives. The cord had sloughed off even with the face of placenta; its attachment was visible; no membranes present, except that covering the placenta; it was about half the usual size, apparently well nourished.

Patient had remained in bed twenty-eight days; I saw her every day, and during that time there was no appearance of the *lochia*, or milk. The lochia came on immediately after the expulsion of placenta, and continued four days. The milk appeared on the second day after, but not sufficient to nourish her child. She made a good and speedy recovery, gaining flesh and strength rapidly. Various other medicines were given during the twenty-eight days to relieve minor troubles, but nothing more to induce uterine contractions.

This lady was living at that time in Anderson county, Tenn., near Coal Creek, and unless she has moved during the last six months, resides there now.

